# Protective effect of aqueous and ethanolic extracts of *Lippia citriodora *Kunth*. *on acrylamide-induced neurotoxicity

**DOI:** 10.22038/AJP.2021.19173

**Published:** 2022

**Authors:** Zahra Tandisehpanah, Amir Foroutanfar, Ali Aziminia, Mahboobeh Ghasemzadeh Rahbardar, Bibi Marjan Razavi, Hossein Hosseinzadeh

**Affiliations:** 1 *School of Pharmacy, Mashhad University of Medical Sciences, Mashhad, Iran*; 2 *Targeted Drug Delivery Research Center, Pharmaceutical Technology Institute, Mashhad University of Medical Sciences, Mashhad, Iran*; 3 *Department of Pharmacodynamics and Toxicology, School of Pharmacy, Mashhad University of Medical Sciences, Mashhad, Iran*; 4 *Pharmaceutical Research Center, Pharmaceutical Technology Institute, Mashhad University of Medical Sciences, Mashhad, Iran*

**Keywords:** Acrylamide, Lippia citriodora, Lemon verbena, Oxidative stress, Apoptosis, Aloysia citrodora

## Abstract

**Objective:**

Acrylamide (ACR) neurotoxicity is induced by different mechanisms such as oxidative stress and apoptosis. Scientific researchs have indicated the antioxidative properties of *Lippia citriodora*. The protective effect of *L. citriodora* aqueous and ethanolic extracts on ACR-induced neurotoxicity was investigated.

**Materials and methods:**

Male Wistar rats were randomly divided into 13 groups: (1) control, (2) ACR (50 mg/kg, i.p.), (3-6) ACR+aqueous extract (12.5, 25, 50, and 100 mg/kg, i.p.), (7-10) ACR+ethanolic extract (12.5, 25, 50, and 100 mg/kg, i.p.), (11) aqueous extract (100 mg/kg), (12) ethanolic extract (100 mg/kg), and (13) ACR+Vitamin E (200 mg/kg, every other day, i.p.). After 11 days, gait score, MDA, and GSH levels in brain cortical tissue were measured. In the *in vitro* test, the viability of PC12 cells (using MTT test), the amount of reactive oxygen species (ROS; using DCFH-DA method), and the protein levels of Bax, Bcl2 and caspase 3 (by western blotting) were measured.

**Results:**

In the *in vitro* study, the IC_50_ for the treatment of PC 12 cells with ACR after 24 hr was 6 mM. ACR decreased cell viability, but increased ROS level, Bax/Bcl-2 ratio, and caspase-3 protein level. Pre-treatment by *L. citriodora* extracts (15-120 µg/ml) ameliorated the toxic effects of ACR on PC12 cells. In the *in vivo* experiment, ACR-induced movement disorders increased MDA but decreased GSH content. The extracts of *L. citriodora* improved ACR toxic effects.

**Conclusion:**

Aqueous and ethanolic extracts of *L. citriodora* were found to reduce ACR-induced neurotoxicity via inhibiting oxidative stress and apoptosis.

## Introduction

Acrylamide (ACR) is a reactive molecule used in a variety of industrial applications such as dye synthesis, wastewater control, soil coagulation, and paper and plastic manufacturing (Ghasemzadeh Rahbardar et al., 2020 ▶; LoPachin, 2004 ▶). Studies have shown that humans might be exposed to ACR through fast food consumption, including coffee, cookies, bread, and fried potatoes heated to temperatures above 120^o^C (Claus et al., 2008 ▶). Investigations have shown that in animals, ACR induces neurotoxicity and reproductive toxicity (Ghasemzadeh Rahbardar et al., 2021 ▶; Shipp et al., 2006 ▶; Tyl and Friedman, 2003 ▶). One of the key processes implicated in ACR-induced toxicity is the buildup of intracellular reactive oxygen species (ROS). In neuroblastoma cells (SH-SY5Y), ACR treatment increased the expression of apoptotic proteins (Sumizawa and Igisu, 2007 ▶). 

Recently, antioxidant agents which are present in plant extracts, have received massive attention from the research community. Lemon verbena or *Lippia citriodora* Kunth. is a member of the Verbenaceae family. Verbascoside and isoverbascoside are the main phenolic substances in *L. citriodora* extract (Etemad et al., 2016 ▶). Because of the lemon-like aroma, the leaves of this plant are utilized in a variety of foods and teas. In folk medicine, lemon verbena has long been used to cure a variety of diseases, including fever, colds, skin infections, and digestive issues. Lemon verbena has also been utilized for its anti-spasmodic, anti-pyretic, and calming properties (Quirantes‐Piné et al., 2010 ▶; Shahhoseini et al., 2014 ▶). Lippia extracts and verbascoside, the most common ingredient, have been shown to have analgesic (Amin et al., 2018 ▶), neuroprotective (Razavi et al., 2017 ▶), antiulcerogenic (Lenoir et al., 2012 ▶), antibacterial (Ghaemi et al., 2007 ▶), anti-fungal, anti-tumor (Cushnie and Lamb, 2005 ▶), anti-proliferative (Lee et al., 2007 ▶), and learning and memory-improving properties (Lee et al., 2006 ▶). Pharmacological studies revealed the antioxidant (Bahramsoltani et al., 2018 ▶; Lenoir et al., 2011 ▶), anti-inflammatory (Sanchez et al., 2013 ▶), and anti-apoptotic (Amin et al., 2018 ▶) properties of this plant.

The protective effects of aqueous and ethanolic extracts of *L. citriodora* on ACR-induced neurotoxicity were assessed in this study by assessing behavioral factors and oxidative stress factors (malondialdehyde (MDA) and glutathione (GSH)) in brain cortical tissue. PC12 cells were employed in the *in vitro* investigation, and the impact of ACR on the viability of these cells, ROS, Bax/ Bcl-2 ratio and caspase 3 level as well as the impacts of *L. citriodora* extracts on ACR toxicity, were assessed. 

## Materials and Methods

**Chemicals and reagents**

ACR and fetal bovine serum (FBS) were purchased from Merck (Darmstadt, Germany), and Gibco (Karlsruhe, Germany), respectively. MTT (3-(4, 5-dimethylthiazol-2-yl)-2, 5-diphenyltetrazolium bromide ) and Dulbecco’s modified Eagle’s medium (DMEM) were obtained from Sigma-Aldrich (Hamburg, Germany). Polyvinylidene fluoride (PVDF) membrane and enhanced chemiluminescence (ECL) western blotting substrate were provided from Bio-Rad (CA, USA) and Thermo Fisher Scientific.

**Plant**

*L. citriodora* leaves were gathered from the surrounding districts of Karaj, Iran (2013/05/04) and identified by herbalists at Mashhad University of Medical Sciences' Department of Pharmacognosy, Faculty of Pharmacy (herbarium No. 12031).

**Preparation of the aqueous extract of the leaves of** ***L. citriodora***

In a 2.5 L glass flask, 200 g powdered plant material was mixed with 2 L boiling water to make aqueous extract of *L. citriodora*. After that, the mixture was heated for 15 min. The solution was then filtered through Whatman No. 1 filter paper and concentrated under vacuum in a rotary evaporator at 46°C. The resulting residues (20 g) were frozen at -20°C until they were used (Portmann et al., 2012 ▶).

**Preparation of the ethanolic extract of the leaves of** ***L. citriodora***

The ethanolic extract of *L. citriodora* was made by macerating 200 g of powdered plant material in 1200 ml of 100% ethanol in a 2.5 L glass flask for 48 hr. The solvent was then evaporated at 30°C after the mixture was filtered using Whatman No. 1 filter paper. The resultant extract (22 g) was frozen at -20°C until needed (Amin et al., 2018 ▶).

***In vitro*** **study**

**Cell culture**

The PC12 cell line was purchased from the Pasteur Institute of Tehran, Iran. PC12 cells were cultured in the RPMI-1640 media supplemented with 10% FBS, 100 U/ml penicillin, and 100 U/ml streptomycin (Gibco, USA). Cells were cultured at 37^o^C in an environment containing 5% CO_2_.

**MTT assay**

To control the condition of cells in culture media, the survival rate, and the status of cells after various chemical treatments, several approaches are used. The MTT test is one of these procedures, which measures a metabolic product to assess and validate cell mitochondrial activity, which is linked to cell viability. In the active mitochondria of living cells, MTT, a yellow tetrazolium salt, is changed to the purple metabolite formazan via the enzyme dehydrogenase. Then, the crystals are dissolved in dimethyl sulfoxide (DMSO), and the light absorption intensity of the dye mixture at 545 and 630 nm is measured using a spectrophotometer. As the number of living cells in the experimental sample decreases, so does the total metabolic activity. This reduction is attributed to the production of purple crystals and reveals the level of mitochondrial activity, and cells viability (Satpute et al., 2008 ▶).

**Measuring intracellular ROS**

The 2,7 dichloro-dihydro-fluorescein diacetate (DCFH-DA) technique was used to evaluate the intracellular ROS levels (Li et al., 2008 ▶). PC12 cells were cultured in 96-well microplates at a density of 4000 cells/well for the experiment. Cells were pretreated for 24 hr with *L. citriodora* aqueous and ethanolic extracts (7.5, 15, 30, 60, and 120 g/ml), then treated with ACR (6 mM), and after overnight incubation, the upper media was separated and the cells were incubated with DCFH-DA (10 M) for 30 min at 37^o^C. Cells were washed in phosphate-buffered saline (PBS) and monitoring dichlorofluorescein (DCF) fluorescence was performed in a microplate reader with an excitation wavelength of 485 nm and an outflow wavelength of 535 nm.

**Western blot analysis**

In brief, cells were pretreated for 24 hr with *L. citriodora* aqueous and ethanolic extracts (30 and 60 g/ml), then treated with ACR (6 mM), and after overnight incubation, cells were lysed in a lysis buffer containing 50 mM Tris-HCl (pH 7.4), 2 mM ethylenediaminetetraacetic acid (EDTA), 2 mM egtazic acid (EGTA), 10 mM NaF, 1 mM sodium orthovanadate (Na_3_VO_4_), 10 mM b-glycerophosphate, 0.2% W/V sodium deoxycholate, 1mM phenylmethylsulfonyl fluoride (PMSF), and a complete protease inhibitor mixture. Bradford assay kit (BioRad, USA) was used to measure the protein content (Chang and Zhang, 2017 ▶). Each adjusted sample was boiled, aliquoted, and stored at -80°C after being mixed with 2X sodium dodecyl sulfate (SDS) blue buffer. Loaded samples were electrophoresed on a 12% SDS-polyacrylamide gel electrophoresis (SDS-PAGE) gel before being blotted to a polyvinylidene fluoride membrane (Bio-Rad, Hercules, CA). The membranes were blocked with 5% non-fat milk powder (skimmed milk) for 2 hr at 37°C and washed with Tris-Buffered Saline and Tween 20 (TBST) three times. Then, the membranes were incubated with rabbit primary polyclonal antibody against Bax (1:1000, Cell Signaling Cat# 2772), rabbit primary monoclonal antibodies against Bcl-2 (1:1000, Cell Signaling Cat# 2870), caspase-3 (1:1000, Cell Signaling Cat# 9665), and rabbit primary monoclonal antibody against β actin (1:1000, Cell Signaling Cat# 3700) for 2 hr on a rocker. After washing, the membranes were incubated with rabbit or mouse horseradish peroxidase-conjugate anti-IgG antibody attached to horseradish peroxidase (1:3000, Cell Signaling Cat# 7074 and 1:3000 Cell Signaling Cat# 7076, respectively) for 2 hr. The peroxidase-coated bands were observed by using enhanced chemiluminescence (Pierce, Rockford, IL). Alliance 4.7 Gel doc (Cambridge, UK) was used to measure bands optical density. UVITEC software was utilized to do the densitometric analysis of protein bands (Cambridge, UK). The levels of the proteins were compared to those of β-actin, which was used as a control protein and had matching bands.

***In vivo*** **study**

**Animals**

Male Wistar rats weighing 240 to 250 g were collected from School of Pharmacy, Mashhad University of Medical Sciences, and kept at 22-25°C under cycles of 12 hr of light and 12 hr of darkness. There was no limit to the use of water and food. All animal tests were done according to the rules of the ethics committee of Mashhad University of Medical Sciences.

**Experimental design**

 Rats were randomized and divided into 13 groups (6 rats in each group) as follows: 

1: control group (normal saline); 2: ACR-treated group (rats treated with ACR 50 mg/kg); groups 3,4,5, and 6: rats received ACR (50 mg/kg) plus aqueous extract of *L. citriodora *(12.5, 25, 50 and 100 mg/kg respectively); groups 7, 8, 9, and 10: rats received ACR (50 mg/kg) plus ethanolic extract of *L. citriodora* (12.5, 25, 50 and 100 mg/kg, respectively); 11: rats received aqueous extract of *L. citriodora* (100 mg/kg); 12: rats received ethanolic extract of *L. citriodora *(100 mg/kg); 13: rats received ACR (50 mg/kg) plus vitamin E (200 IU/kg). Our latest experimental results and other studies have proven that the best dose of ACR that induces - toxicity is 50 mg/kg (Esmaeelpanah et al., 2018; Mehri et al., 2014). All treatments were injected intraperitoneally once daily for 11 days except vitamin E which was given every other day. Each protective dose was injected 30 min before ACR injection.

**Behavioral examination**

The behavioral index (gait score) was tracked after the treatment period ended. The rats were located in a clear box (85×85×40 cm) and their movements were observed for three min. The behavioral results were graded on a four-point scale, as follows: 1: Steps and walking are normal; 2: Steps and walking are minimally affected (foot splay and slight hind limb weakness); 3: Steps and walking are moderately affected (foot splay with a limb spread during ambulation); and 4: Steps and walking are extremely influenced (foot splay, strong hind limb weakness, dragging hind limbs, and disability to rear) (LoPachin, 2005 ▶).

**Collecting tissue samples**

Animals in each group were decapitated once the behavioral tests were completed. The cerebral cortex was promptly removed from the hemispheres at the level of the medial longitudinal fissure, rinsed in cold saline solution (0.9%), placed in 5 mL micro-tubes, frozen in liquid nitrogen, and kept at -80°C (Tomassoni et al., 2020 ▶).

**Biochemical assay**

**Lipid peroxidation measurement**

MDA is a marker of lipid peroxidation, and higher MDA levels indicate more lipid peroxidation. MDA produces a pink color in acidic media when combined with TBA, which has the highest color absorption at 532 nm (Ohkawa et al., 1979 ▶). For creating a cool homogeneous solution, 10% of the cerebral cortex was dissolved in cold 1.15% KCl. Then, 3 ml phosphoric acid 1% and 1 ml TBA 0.6 % were mixed with 0.5 ml homogenized tissue simultaneously. The tubes were then boiled for 45 min in hot water. Then, the tubes were removed and cooled. Then, n-butanol (4 ml) was added and tubes were vortexed for one minute. After that, the mixtures were centrifuged for 10 min at 3000 g. After centrifugation, the supernatant was collected and absorbance was measured at 532 nm. Based on a standard curve drawn for MDA (concentration range 0-100 nmol/ml), the levels were calculated as nmol/g tissue (Uchiyama and Mihara, 1978 ▶).

**Determination of GSH content**

In this method, free sulfhydryl groups react with 5,5-dithio-bis-(2-nitrobenzoic acid; DTNB), producing a colorful complex with the maximum absorbance observed at 412 nm. GSH was utilized to draw the standard curve. GSH concentrations are reported as nmol/gr of tissue. The tissue was mixed with 1:1 trichloroacetic acid (TCA, 10%) and centrifuged at 3000 g for 10 min. Then, 2.5 mL of phosphate buffer (pH 8) and 0.1 M PBS were added to the supernatant. The absorbance at 412 nm was measured after addition of 0.5 ml DTNB to the sample (Moron et al., 1979 ▶). 

**HPLC analysis of the aqueous and ethanolic extracts of** ***L. citriodora***

The fingerprint of aqueous and ethanolic extracts of *L. citriodora* was characterized by high-pressure liquid chromatography (HPLC). HPLC analysis was performed on an analytical Knauer HPLC system equipped with a k-1001 Knauer pump (flow rate of 0.8 ml/min), with a C18 hyper sail column, and a k-2600 Knauer UV detector. Chromatographic separation was carried out by injecting the sample onto the C18 hyper sail column (4.6×150 mm). An isocratic elution was accomplished with the solvent system methanol: water with a gradient elution (0 min, 10:90; 8 min, 100:0; 12 min, 100:0; 14 min, 10:90; 16 min, 10:90) at a flow rate of 0.8 ml/min. The peaks were observed at 254 and 280 nm. The volume of injection was 20 μl.

**Statistical analysis**

To analyze the gait abnormalities, the nonparametric Kruskal–Wallis test followed by Dunn's multiple comparison test, was used. The results of the lipid peroxidation test, the GSH content assay, and the western blot test are shown as mean±SD. For statistical analysis, one-way ANOVA with *post hoc* test Tukey, was performed. Differences were considered statistically significant when p<0.05.

## Results

**Effect of ACR, and aqueous and ethanolic extracts of** ***L. citriodora*** **on PC12 cells viability**

**Figure 1:**
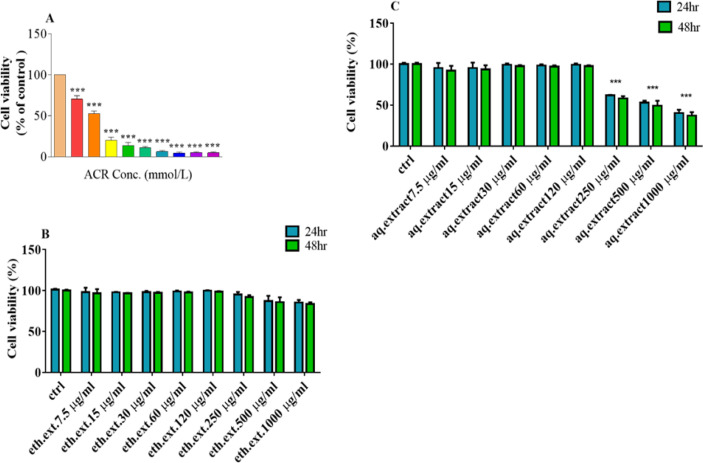
A: Viability of PC12 cells after exposure to acrylamide (ACR) for 24 hr. B: Viability of PC12 cells after exposure to ethanolic extract of *L. citriodora* (eth.ext) for 24 and 48 hr. C: Viability of PC12 cells after exposure to aqueous extract of *L. citriodora* (aq.ext) for 24 and 48 hr. Cell viability was determined by the MTT test. Data are expressed as mean±SD of four separate experiments. ANOVA and Tukey-Kramer post-test were used for statistical analysis. ***p<0.001 vs. control. Ctrl: control; ACR: acrylamide; aq. ext: aqueous extract; eth. ext: ethanolic extract

As shown in Figure 1A, different concentrations of ACR significantly decreased cell viability in a concentration-dependent manner after 24 hr of exposure. The ACR IC_50_ for the cells after 24 h was 6 mM. The cell viability was determined by the MTT test.

Results of exposure of the PC12 cells to different concentrations of ethanolic and aqueous extracts (7.5-1000 µg/ml) are shown in Figures 1B and C. There was no meaningful change in cell viability between different concentrations of ethanolic extract (7.5-1000 µg/ml) and the control, while, three concentrations of aqueous extract (250, 500, and 1000 µg/ml) significantly reduced cell viability after 24 and 48 hr compared to the control (p<0.001; Figure 1B and C) 

**Effect of aqueous and ethanolic extracts of** ***L. citriodora*** **on ACR-induced cytotoxicity in PC12**

**Figure 2:**
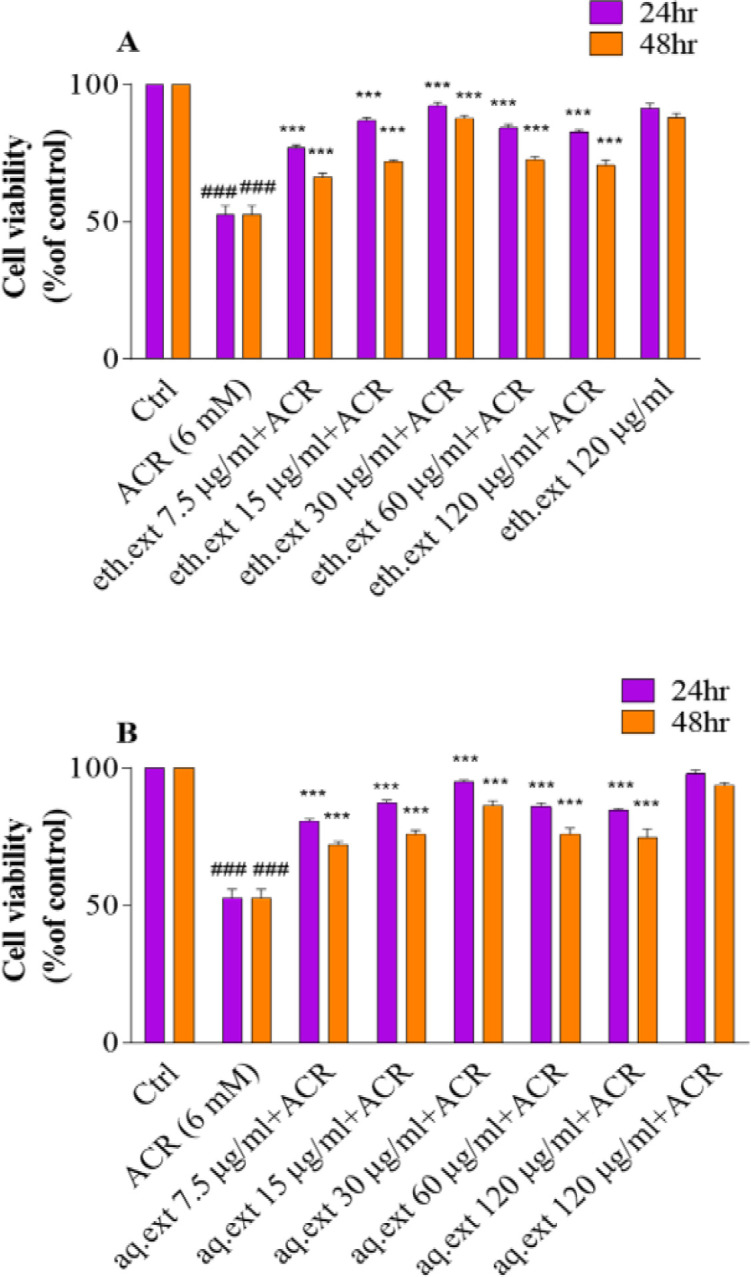
A: Effects of ethanolic extract of *L. citriodora *(eth.ext) on ACR-induced cytotoxicity in PC12 cells after 24 and 48 hr. B: Effects of aqueous extract of *L. citriodora* (aq.ext) against ACR-induced cytotoxicity in PC12 cells after 24 and 48 hr. Cells were pretreated with different concentrations of aqueous and ethanolic extracts of *L. citriodora *(7.5–120 µg/ml) before exposure to 6 mM of ACR. Data are expressed as mean±SD of four separate experiments. ANOVA and Tukey-Kramer post-test were used for statistical analysis. ###p<0.001 vs. control and ***p<0.001 vs. ACR. Ctrl: control; ACR: acrylamide; aq. ext: aqueous extract; eth. ext: ethanolic extract

As shown in Figure 2, ACR-induced cytotoxicity in PC12 cells significantly reduced cell viability after 24 and 48 hr in comparison with the control group (52.65±3.25 vs 100.00±0.00) (p<0.001). On the other hand, co-treatment with various concentrations of aqueous or ethanolic extracts of *L. citriodora* (7.5-120 µg/ml) remarkably increased cell viability in comparison with the ACR-treated cells (p<0.001).

**Effect of aqueous and ethanolic extracts of** ***L. citriodora*** **on ROS accumulation induced by ACR in PC12 cells**

**Figure 3:**
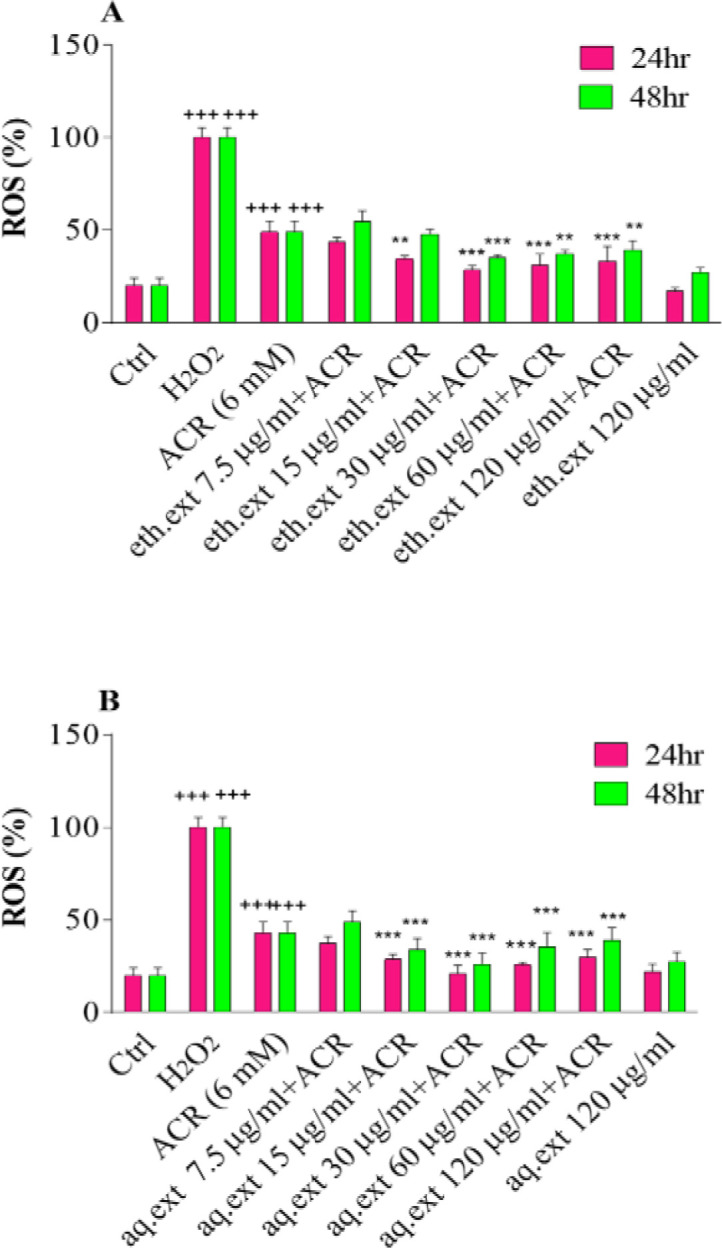
A: Effects of ethanolic extract of *L. citriodora* (eth.ext) on ROS production in PC12 cells after 24 and 48 hr exposure. B: Effects of aqueous extract of *L. citriodora* (aq.ext) on ROS production in PC12 cells after 24 and 48 hr of exposure. Cells were pretreated with different concentrations of aqueous and ethanolic extracts of *L. citriodora* (7.5–120 µg/ml) before exposure to 6 mM of ACR. Data are expressed as mean±SD of four separate experiments. ANOVA and Tukey-Kramer post-test were used for statistical analysis. ###p<0.001 vs. control and ***p<0.001 and **p<0.01 vs. ACR. H_2_O_2_ was used as a positive control. Ctrl: control; ACR: acrylamide; aq. ext: aqueous extract; eth. ext: ethanolic extract

ACR exposure significantly increased intracellular ROS in PC12 cells compared to the control (48.96±5.99 vs 19.82±4.18) (p<0.001). Pretreatment of cells with various concentrations of aqueous and ethanolic extracts of *L. citriodora* (15, 30, 60, and 120 µg/ml) markedly inhibited ROS production as compared to the ACR-treated cells (p<0.001 and p<0.01) (Figures 3A and B). 

**Effect of aqueous and ethanolic extracts of** ***L. citriodora*** **on expression of the proteins involved in apoptosis**

**Figure 4:**
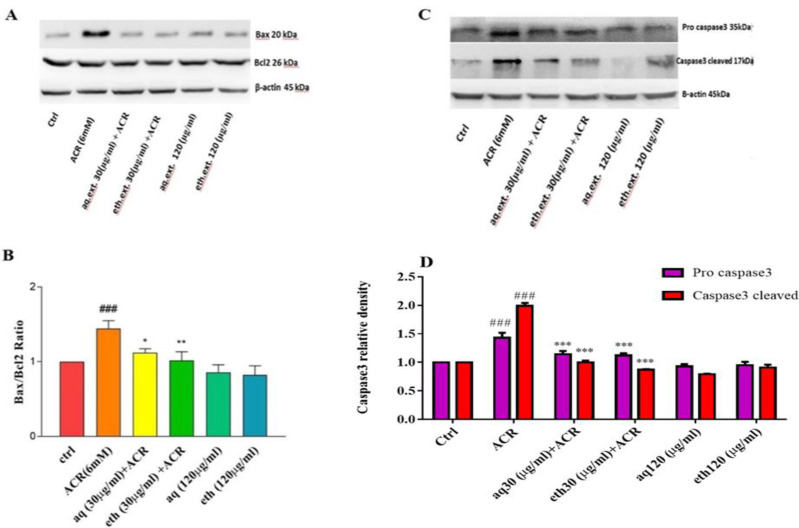
Effect of ethanolic (eth.ext) and aqueous extract (aq.ext) of *L. citriodora* on the protein expression induced by ACR in PC12 cells. (A) Bax and Bcl-2 specific band proteins that were monitored by western blotting. (B) Bax/Bcl-2 ratio was determined using densitometric analysis. (C) Pro-caspase-3 and cleaved caspase-3 specific band proteins that were monitored by western blotting. (D) Pro-caspase-3 and cleaved caspase-3 were determined using densitometric analysis. Values are presented as mean±SD, (n=4). ANOVA and Tukey-Kramer Post-test were used for statistical analysis. ###p<0.001vs. the control, and **p<0.01 and *p<0.05 vs. the ACR group. β-actin was used as an internal control. Ctrl: control; ACR: acrylamide; aq. ext: aqueous extract; eth. ext: ethanolic extract

In this study, aqueous and ethanolic extracts at 30 µg/ml showed more desirable effects compared to the other concentrations. Hence, this concentration was chosen for anti-apoptotic evaluation. As shown in Figure 4B, ACR increased the level of Bax protein without significant alterations in the level of Bcl-2 protein. The Bax/Bcl-2 ratio significantly increased compared to the control (1.44±0.10 vs 1.00±0.00) (p<0.001).

Moreover, the amount of caspase-3 protein (pro and cleaved) elevated meaningfully in the ACR-treated cells in comparison with control (Pro: 1.43±0.90 vs 1.00±0.00; and cleaved: 1.99±0.55 vs 1.00±0.00) (p<0.001, Figure 4D).

On the other side, pretreatment with aqueous and ethanolic extracts of *L. citriodora *reduced significantly the Bax protein level which leads to decreased Bax/Bcl-2 ratio compared to the ACR-treated cells (Aqueous: 1.12±0.05; and ethanolic: 1.017±0.011 with p<0.05 and p<0.01, respectively, Figure 4B). Also, exposure of the PC12 cells to aqueous and ethanolic extracts of *L. citriodora* led to reduced expression of caspase-3 compared to the ACR-treated cells (Aqueous: 1.14±0.60 for Pro and 1.00±0.34 for cleaved and ethanolic: 1.12±0.49 for Pro and 0.87±0.184 for cleaved) (p<0.001, Figure 4D).

**Effect of ACR as well as aqueous and ethanolic extracts of** ***L. citriodora*** **on** **the behavioral index (gait scores) in rats **

**Figure 5:**
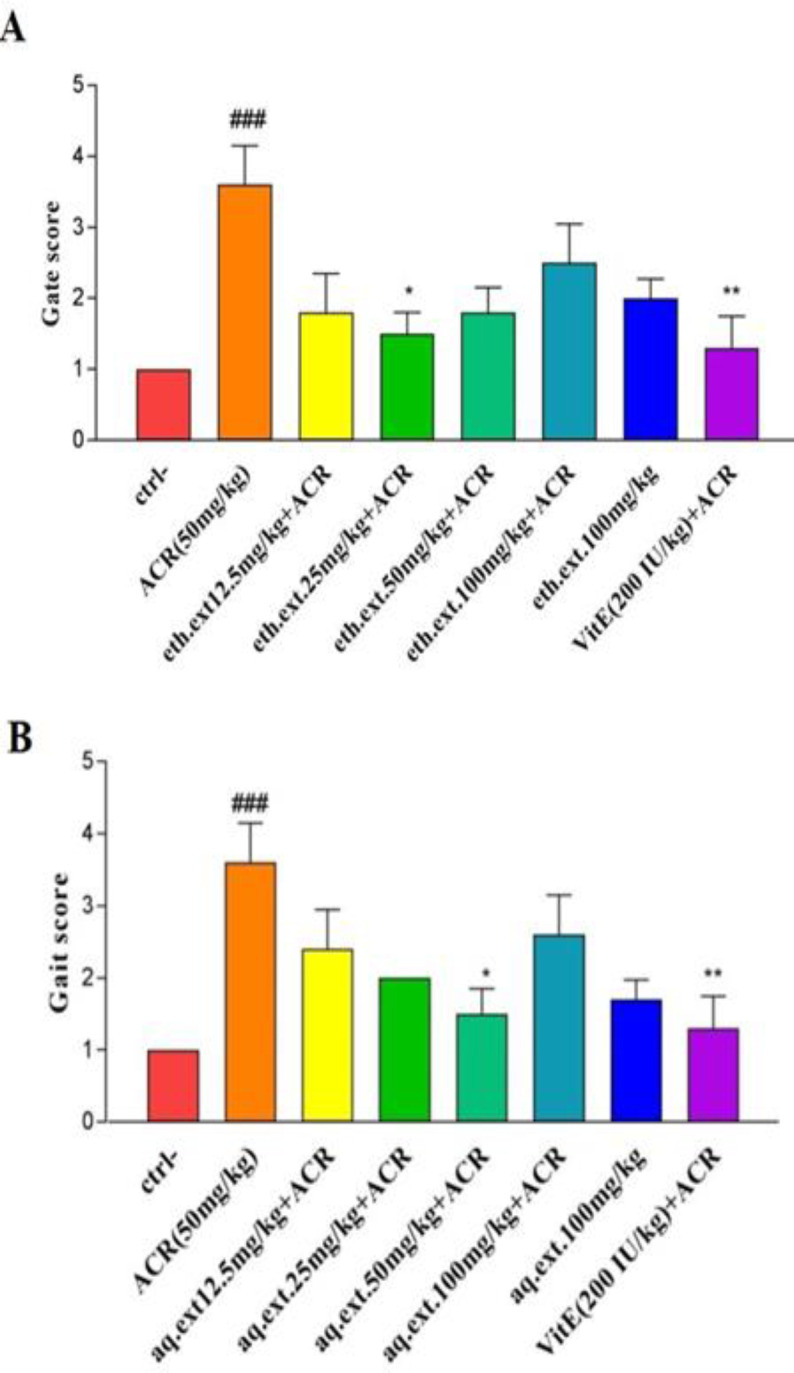
A: Effect of ethanolic extract of *L. citriodora* (eth.ext) on motor disorders induced by ACR. B: Effect of aqueous extract of *L. citriodora *(aq.ext) on motor disorders induced by ACR. Values are presented as median with interquartile range, (n = 6). ###p<0.001 vs. the control and ^*^p<0.05 and ^**^p<0.01 vs. the ACR group. Ctrl: control group, ACR: Acrylamide and Vit E: vitamin E. Ctrl: control; ACR: acrylamide; aq. ext: aqueous extract; eth. ext: ethanolic extract; Vit E: vitamin E

Administration of ACR for 11 days caused significant weight loss and gait score abnormality in rats compared to the control group (p<0.001), while co-treatment with aqueous (50 mg/kg) or ethanolic (25 mg/kg) extracts of *L. citriodora *improved gait abnormalities significantly in comparison to the ACR group (p<0.05, Figure 5). 

**Effect of aqueous and ethanolic extracts of** ***L*** ***.*** ***citriodora*** **on lipid peroxidation and GSH content induced by ACR in cerebral cortex tissue**

**Figure 6:**
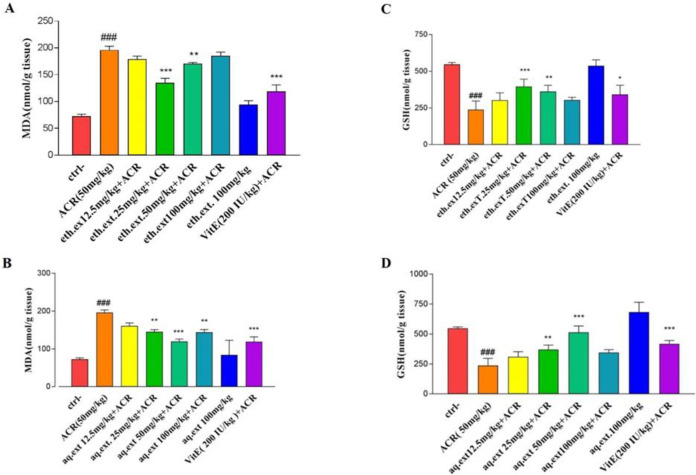
A: Effect of ethanolic extract of *L. citriodora* (eth.ext) on ACR-induced lipid peroxidation in cerebral cortex tissue. B: Effect of aqueous extract of *L. citriodora *(aq.ext) on ACR-induced lipid peroxidation in cerebral cortex tissue. C: Effect of ethanolic extract of *L. citriodora* (eth.ext) on GSH content in cerebral cortex tissue. D: Effect of aqueous extract of *L. citriodora* (aq.ext) on GSH content in cerebral cortex tissue. Values are presented as mean±SD (n = 6). ANOVA and Tukey-Kramer post-test was used for statistical analysis. ###p<0.001 vs. control. *p<0.05, **p<0.01 and ***p<0.001 vs. ACR group. Ctrl: control; ACR: acrylamide; aq. ext: aqueous extract; eth. ext: ethanolic extract; Vit E; vitamin E

The injection of ACR for 11 days led to a meaningful increase in MDA level as compared to the control group (196.14±7.06 vs 72.53±3.80 nmol/g tissue) (p<0.001. Figure 6). On the other hand, co-administration of aqueous or ethanolic extracts showed desirable effects on ACR-induced toxicity, in which the aqueous extract at doses of 25 mg/kg (145.11±5.96 nmol/g tissue) (p<0.01) , 50 mg/kg (119.54±6.50 nmol/g tissue) (p<0.001), and 100 mg/kg (144.15±7.17 nmol/g tissue) (p<0.01) and ethanolic extracts at doses of 25 mg/kg (134.82±8.60 nmol/g tissue) (p<0.001) and 50 mg/kg (170.80±2.05 nmol/g tissue) (p<0.01) significantly decreased lipid peroxidation in cerebral cortex compared to the ACR-treated rats (Figure 6B and 6A).

 According to Figures 6C and D, ACR administration caused a significant decline in GSH contents in brain tissue in comparison with the control group (238.25±59.21 vs 547.00±12.91 nmol/g tissue) (p<0.001), while, co-treatment with 25 mg/kg (370.80±37.02 nmol/g tissue) (p<0.01) and 50 mg/kg (514.75 ± 52.52 nmol/g tissue) (p<0.001) doses of aqueous and 25 mg/kg (395.75±50.22 nmol/g tissue) (p<0.001) and 50 mg/kg (361.30±42.57 nmol/g tissue) (p<0.01) doses of ethanolic extracts led to meaningful increases in cerebral glutathione contents as compared to the ACR-treated rats. 

**The HPLC fingerprints**

**Figure 7:**
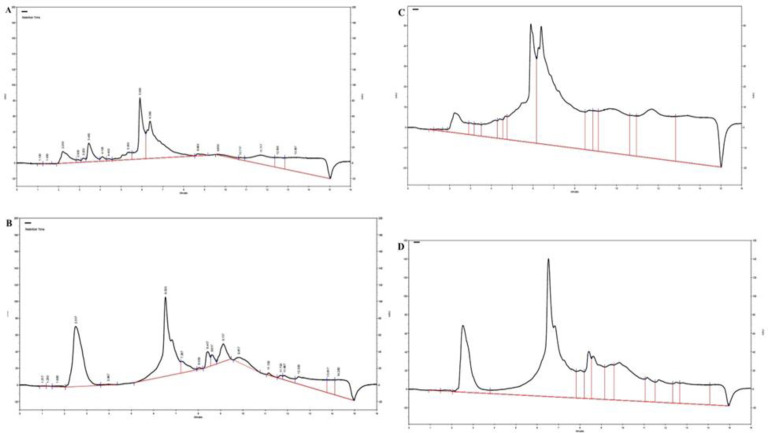
A: HPLC fingerprint of the aqueous extract of *L. citriodora* at the wavelength of 254 nm. B: HPLC fingerprint of the ethanolic extract of *L. citriodora* at the wavelength of 254 nm. C: HPLC fingerprint of the aqueous extract of *L. citriodora* at the wavelength of 280 nm. D: HPLC fingerprint of the ethanolic extract of *L. citriodora* at the wavelength of 280 nm

HPLC fingerprints of the aqueous extract of *L. citriodora* displayed major peaks at the wavelength of 254 nm, for the retention times (minute) of 2.20, 3.45, 5.90, and 6.38 (Figure 7A). Likewise, HPLC fingerprints of the ethanolic extract of *L. citriodora* presented major peaks at the wavelength of 254 nm, for the retention times (minute) of 2.51, 6.53, 8.41, 8.61, and 9.11 (Figure 7B). HPLC fingerprints of the aqueous and ethanolic extract of *L. citriodora* at the wavelength of 280 nm are shown in Figures 7C and D.

## Discussion

In this study, the desirable effects of aqueous and ethanolic extracts of *L. citriodora* against ACR-induced neurotoxicity and cytotoxicity were evaluated. 

Our data demonstrated that ACR (6 mM) led to a significant reduction in cell viability and enhanced the accumulation of intracellular ROS while pretreatment with various concentrations of aqueous and ethanolic extracts of *L. citriodora* showed protective effects against cytotoxicity induced by ACR. It was also indicated that the intracellular ROS production was reduced meaningfully by pretreatment with these extracts at different concentrations; both extracts at the concentration of 30 µg/ml were more effective than the other concentrations. Several studies have proven that ACR caused cytotoxicity and enhanced ROS accumulation (Esmaeelpanah et al., 2018 ▶). In Mehri *et al*. study, the viability of PC12 cells significantly reduced after 24 and 48 hr exposure to ACR and this toxicant increased the ROS production meaningfully (Mehri et al., 2012 ▶). 

*L. citriodora* is one of the plants with high phenolic and flavonoid contents. Phenylpropanoids, mainly verbascoside, are present in *Lippia *extracts in high amounts (Carrera‐Quintanar et al., 2012 ▶). The extract of this plant has free-radical scavenging properties and increased cell viability in H_2_O_2_-induced toxicity (Yoo et al., 2008). In López *et al* study, both *L. citriodora* and verbascoside declined the intracellular ROS generation and improved the mitochondrial membrane potential in high-glucose-induced hypertrophic adipocytes (Herranz-López et al., 2015 ▶). Oxidative stress-induced by ACR was considered one of the mechanisms that induced apoptosis (Rodríguez-Ramiro et al., 2011 ▶). 

The balance between the anti-apoptotic proteins and the apoptotic initiators plays a major role in the process of apoptosis. Although Bax protein is the initiator of the apoptotic process, Bcl-2 is an apoptotic inhibitor and has anti-apoptotic properties (Elmore, 2007 ▶). Caspase-3 is known as the downstream effector which has a critical role in the apoptosis process (Tabeshpour et al., 2019 ▶). In this study, ACR induced cellular apoptosis significantly. Though Bcl-2 protein expression did not alter, the amount of Bax protein increased meaningfully. Hence, the ratio of Bax/Bcl-2 which plays the principal role in the development of the apoptosis process significantly elevated in PC12 cells. Furthermore, exposure to ACR caused a significant elevation in caspase-3 protein level. Other studies have indicated that the Bax/Bcl-2 ratio significantly increased due to exposure to ACR (Chen et al., 2009 ▶; Esmaeelpanah et al., 2018 ▶). On the other hand, pretreatment of PC12 cells by aqueous and ethanolic extracts of *L. citriodora *(30 µg/ml) improved apoptotic conditions and reduced the Bax/Bcl-2 ratio significantly. We also observed a similar effect in caspase-3 levels where the aqueous and ethanolic extracts declined pro and cleaved caspase-3 meaningfully. Similar to our study, *L. citriodora* ethanolic extract (100 and 200 mg/kg) reduced the Bax/Bcl-2 ratio in the rodent spinal cord (Amin et al., 2018 ▶). 

The intraperitoneal injection of ACR (50 mg/kg) for 11 days has been chosen for the induction of neurotoxicity in rats according to the previous studies (Esmaeelpanah et al., 2018 ▶; Mehri et al., 2014 ▶). Results showed that ACR induced severe gait abnormality in rats. On the other side, co-treatment with aqueous or ethanolic extracts of *L. citriodora *showed a desirable effect on motor disorders. In Mehri *et al* study, injection of ACR (50 mg/kg) for 11 days caused significant weight loss and movement disability in rats. However, co-treatment by crocin at the dose levels of 25 and 50 mg/kg relieved gait abnormalities (Mehri et al., 2012 ▶). The probable mechanism involved in gait abnormality is oxidative damage. Hence, the protective effect of aqueous and ethanolic extracts of *L. citriodora *may be due to its antioxidant activities. Similar protective effects have been indicated with the bioflavonoid in other behavioral models (Esmaeelpanah et al., 2018 ▶). 

Our data demonstrated that exposure to ACR leads to oxidative stress in the cerebral cortex. Although the MDA level as a marker of lipid peroxidation, increased meaningfully, the reduced glutathione content decreased significantly in ACR-treated rats. Several studies have proven that ACR-induced neurotoxicity through oxidative damage (Mehri et al., 2014 ▶; Sumizawa and Igisu, 2007 ▶). In this regard, Prasad *et al.* indicated that ACR increased oxidative stress parameters including MDA, nitric oxide, and ROS in the cortex and cerebellum (Prasad, 2013 ▶). In contrast, our results disclosed that co-treatment by aqueous or ethanoic extracts of *L. citriodora* relieved oxidative stress conditions. It not only compensated for the lack of GSH contents but also diminished the MDA levels in the cerebral cortex. These desirable effects were due to the high antioxidant potential of verbascoside (Amin et al., 2018 ▶). Our results are in agreement with the Qiusheng *et al.* study in which, verbascoside was able to abate the oxidative stress induced by heroin in rodent brain tissue (Qiusheng et al., 2005 ▶). 

Although both ethanolic and aqueous extracts of *L. citriodora *showed protective activity against ACR-induced neurotoxicity, it seems that the phenylpropanoids verbascoside and isoverbascoside were more presented in the ethanolic extract more than aqueous extract (Funes et al., 2009 ▶). Antioxidants such as vitamin E, GSH and some phytochemicals can show antioxidant and pro-oxidant dual effects. Their pro-oxidant effects are related to micro-environmental circumstances such as relative antioxidant capacity, concentration, bioavailability, presence of transition metals, type of cell line (e.g. normal or cancerous) and different laboratory conditions (Esmaeelpanah et al, 2018 ▶).

In conclusion, the results of the current study suggest that both ethanolic and aqueous extracts of *L. citriodora *exhibit antioxidant and anti-apoptotic activity. The mechanism involved in the beneficial effect of these extracts may be related to inhibition of ACR-increased intracellular ROS accumulation *in vitro* and elevation of ACR-reduced GSH content *in vivo*.

## Conflicts of interest

The authors have declared that there is no conflict of interest.
